# Neurotrophic Keratopathy after Trigeminal Nerve Block for Treatment of Postherpetic Neuralgia

**DOI:** 10.1155/2018/6815407

**Published:** 2018-05-31

**Authors:** Aya Kodama-Takahashi, Koji Sugioka, Tomoko Sato, Koichi Nishida, Keiichi Aomatsu, Masahiko Fukuda, Yoshikazu Shimomura

**Affiliations:** Department of Ophthalmology, Kindai University Faculty of Medicine, Osaka-Sayama City, Japan

## Abstract

**Purpose:**

To report a case of persistent corneal epithelial defect that had occurred after a trigeminal nerve block.

**Case Presentation:**

A 75-year-old female had suffered from postherpetic neuralgia for 8 years. She underwent Gasserian ganglion block surgery and noticed declining visual acuity in the right eye on the following day. She presented with severe hyperemia and corneal epithelial defects in the right eye and experienced remarkable reduction of sensitivity in the right cornea. She was diagnosed with neurotrophic keratopathy. Ofloxacin eye ointment and rebamipide ophthalmic suspension ameliorated the corneal epithelial defects but superficial punctate keratopathy, corneal superficial neovascularization, and Descemet's fold persisted. Although the epithelial defects occasionally recurred, the corneal sensation and epithelial defects, Descemet's fold, and corneal superficial neovascularization all improved around 5 months after trigeminal nerve block. The HRT II Rostock Cornea Module (RCM) could not detect any corneal subbasal nerve fibers at postoperative 4 months; however, it could detect them at postoperative 6 months.

**Conclusions:**

As the nerve block effect wore off, the corneal subbasal nerve fibers slowly regenerated. As the corneal sensation improved, the corneal epithelial defects and superficial neovascularization also improved. The HRT II RCM appeared useful for observing loss and regeneration of the corneal subbasal nerve fibers.

## 1. Introduction

The corneal epithelium is the outermost layer of the cornea and one of its important roles is to act as a barrier that protects the eye against injuries from the outside world. By receiving pain signals, the first branch of the trigeminal nerve in the cornea can sense foreign bodies and injuries at a very early stage. At the same time, neurotrophic factors such as substance P (SP) derived from the sensory nerves work with growth factors, cytokines, and extracellular matrix in a complex and coordinated manner and participate greatly in maintaining the homeostasis and wound healing of the corneal epithelium [1, 2].

Postherpetic neuralgia refers to the pain that remains after herpes zoster is resolved. When it resists oral analgesic medicine, postherpetic neuralgia is sometimes treated with a trigeminal nerve block. It is well known that trigeminal nerve block results in trigeminal nerve damage and subsequent NK [3, 4]. However, to our knowledge, there are no reports of a case so closely observed: we observed the process in which the blocked corneal nerve was restored within several months after disappearance of subbasal nerve fibers due to the trigeminal nerve block as well as the accompanying improvement of corneal findings.

In this study, we report a rare case of corneal epithelial defects that occurred on the following day of a trigeminal nerve block for postherpetic neuralgia, and the process of loss and regeneration of the corneal subbasal nerve fibers was observed using the Rostock Cornea Module of the Heidelberg Retina Tomograph II (RCM HRT II, Heidelberg Engineering, Carlsbad, CA).

## 2. Case Report

The subject was a 75-year-old woman who had suffered from postherpetic neuralgia for 8 years. In December 2016, she underwent a Gasserian ganglion nerve block at the Department of Anesthesiology of our hospital. Gasserian ganglion nerve block was performed to treat the first branch neuralgia of the trigeminal nerve in the right at the point which is 2.5 cm lateral to the right angle of the mouth, following a straight line directed toward the pupil. A needle was inserted under guidance of X-ray imaging on the monitor display. When the tip of the guiding needle reached the foramen ovale, the location of the needle tip was confirmed to be slightly inside from the median in the frontal view. Next, after confirming dysesthesia, 2% xylocaine was injected. And then loss of sensation was confirmed in the V1 and V2 regions. After injecting 99.5% ethanol (0.2 mL), radiofrequency thermocoagulation was added continuously for 180 seconds at 90°C.

On the following day, the patient noticed a sudden visual acuity (VA) decline and hyperemia in the right eye. She visited a neighborhood ophthalmologist and was diagnosed with conjunctival hyperemia, corneal epithelial defect, and Descemet's folds in the right eye. Because no improvement was achieved, she visited our hospital two days later. The initial VA was 0.03 (n.c.) in the right eye. A slit-lamp examination revealed severe conjunctival hyperemia all around the periphery, remarkable corneal superficial neovascularization, especially, at 11 to 5 o'clock, corneal epithelial defects of the size approximately 3 × 4 mm, Descemet's membrane folds, and mild stromal edema were observed; however, no signs of inflammation were seen in the anterior chamber. The patient did not experience any eye pain and corneal sensitivity measured with Cochet-Bonnet esthesiometer was <10 mm in the right eye and 60 mm in the left, showing remarkable decline of corneal sensitivity in the right. She was diagnosed with NK stage 2 (Table 1).

**Figure 1:**
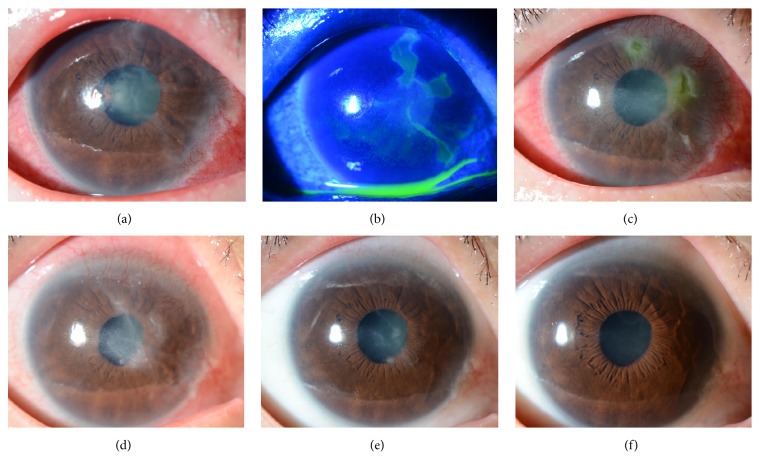
Slit-lamp images of the right eye showing the clinical course.(a) One week after the trigeminal nerve block, corneal epithelial defects, severe conjunctival hyperemia all around the periphery, cloudy and irregular corneal epithelium and Descemet's membrane folds, and mild stromal edema were noted. (b) One week after the trigeminal nerve block, the corneal epithelial defects were shown using fluorescein staining. (c) One month after the nerve block, PED were noted. Severe conjunctival hyperemia, very severe corneal superficial neovascularization at 11 to 5 o'clock, and corneal stromal edema in the central cornea were also observed. (d) Two months after the trigeminal nerve block, the epithelial defects were improved but SPK, corneal superficial neovascularization at 11 to 5 o'clock, irregularity of corneal epithelium, and stromal scarring in the central cornea remained. (e) Five months after the nerve block, slight irregularity of corneal epithelium and stromal scarring in the central cornea were observed. Corneal superficial neovascularization showed signs of improvement although SPK remained. (f) Six months after the nerve block, SPK and conjunctival hyperemia, corneal superficial neovascularization, and corneal stromal edema were all improved.

Treatment was started with 0.3% ofloxacin ophthalmic ointment (Tarivid® ophthalmic ointment 0.3%; Santen Pharmaceutical Co., Ltd., Osaka, Japan) twice daily and rebamipide ophthalmic suspension (Mucosta® ophthalmic suspension unit dose 2%; Otsuka Pharmaceutical Co., Ltd., Tokyo, Japan) 4 times daily. The corneal epithelial defects were gradually alleviated at 1 week after ganglion nerve block (Figures 1(a) and 1(b)).

**Figure 2:**
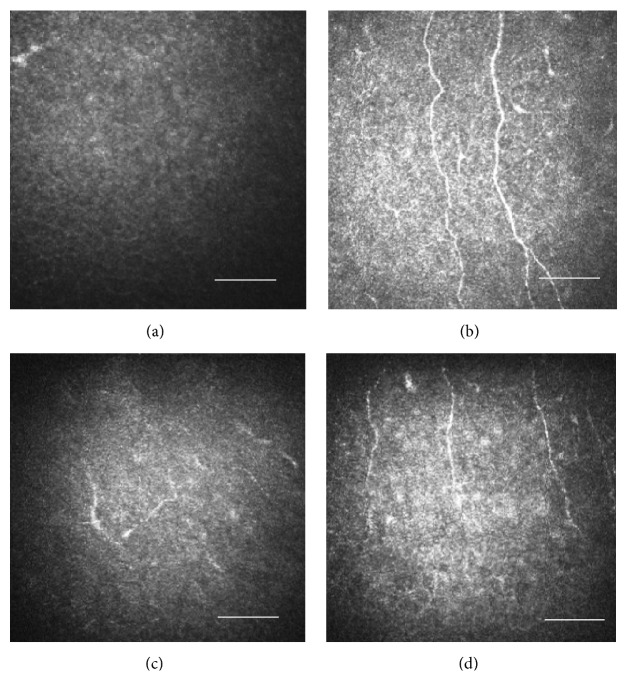
Evaluation of the subbasal nerve fibers in the cornea with the HRT II RCM. Size bar = 100 *μ*m. (a) Four months after the nerve block, the subbasal nerve fibers were hardly observed in the right eye. (b) Four months after the nerve block, the subbasal nerve fibers were observed in the fellow eye. (c) Six months after the nerve block, regenerated subbasal nerve fibers were observed starting from the periphery of the right cornea, although they appeared small and short. (d) Six months after the nerve block, subbasal nerve fibers were observed in the fellow eye.

Because severe conjunctival hyperemia persisted, we added fluorometholone ophthalmic suspension (fluorometholone® ophthalmic suspension unit dose 0.1%; Santen Pharmaceutical Co., Ltd.) 3 times daily. At 1 month after ganglion nerve block (Figure 1(c)) severe conjunctival hyperemia, very severe corneal superficial neovascularization at 11 to 5 o'clock, persistent corneal defects with smooth and rolled edges, and stromal swelling in the central cornea occurred. At 2 months (Figure 1(d)), superficial punctate keratopathy (SPK), conjunctival hyperemia, corneal superficial neovascularization at 11 to 5 o'clock, irregularity of corneal epithelium, stromal scarring in the central cornea, and the right corneal sensation of 10 mm remained. Evaluation with the HRT II RCM was made in April 2017 and no corneal subbasal nerve fibers were observed in the right eye (Figures 2(a) and 2(b)). Five months after the trigeminal nerve block, slight irregularity of corneal epithelium and stromal scarring in the central cornea were observed; however, conjunctival hyperemia and corneal superficial neovascularization were overall improving and the corneal sensation in the right eye also recovered to about 50 mm although SPK remained (Figure 1(e)). At this point, corneal epithelial defect was overall improving; therefore, ofloxacin eye ointment was stopped. Rebamipide and fluorometholone eye drops were continued and the patient was followed up. According to the patient, she had regained the sense of touch on the cheek. Corneal erosion sometimes recurred but it recovered in about a week. Six months after the trigeminal nerve block, the corneal sensation in the right eye was 60 mm. SPK and conjunctival hyperemia, corneal superficial neovascularization, and corneal stromal edema were all ameliorated (Figure 1(f)). Therefore, fluorometholone eye drops were tapered off and only rebamipide eye drops were continued. In addition, at this point, regenerated corneal subbasal nerve fibers in the right eye were detected by the HRT II RCM although they appeared to be smaller and thinner than those in the fellow eye (Figures 2(c) and 2(d)).

## 3. Discussion

We experienced a rare case of NK that gradually ameliorated as the effects of the trigeminal ganglion block for postherpetic neuralgia wore off. Although intractable corneal epithelial defects occurred on the next day of the nerve block, corneal sensation, corneal epithelial defects, corneal superficial neovascularization, and corneal stromal edema were improved as the effects of the nerve block subsided.

Various causes for NK have been reported [3]. Herpetic infection, diabetic keratopathy, and brain tumor can all be the underlying factors for corneal defects with a prominent decline in corneal sensation. SPK, corneal erosion, persistent corneal epithelial defect (PED), corneal ulcer, corneal neovascularization, decreased corneal endothelial cells, and corneal stromal edema are the clinical findings associated with NK [3–5]. In the present case, corneal epithelial defects and corneal endothelial disorders occurred on the following day of the trigeminal ganglion block and persisted for several months until the corneal sensation recovered. This observation demonstrated that the sensory branch of the trigeminal nerve in the cornea plays an important role in maintaining the homeostasis of the cornea and the ocular surface.

Using a confocal microscope to depict the corneal subbasal nerve fibers has been reported previously in a study on NK [6]. In this study, we used the HRT II RCM to observe the corneal subbasal nerve fibers during the process of ocular recovery. Six months after the nerve block, the nerve fibers could be depicted and the regeneration of the subbasal nerve fibers was confirmed. The HRT II RCM appeared useful for diagnosis and observation during follow-up. After approximately five months from the trigeminal nerve block, corneal erosion sometimes reoccurred. Although it recovered within a week or so, the vulnerable adhesion of the corneal epithelium became clear.

Even now no definitive treatments are available for NK or permanently damaged trigeminal nerve [3, 4]. Currently, oil eye ointment, therapeutic contact lens, and forced eyelid closure are used to protect the corneal epithelium. Reportedly, fibronectin instillation, SP + insulin-like growth factor-1 (IGF-1) derived from the trigeminal nerve [7–9], and a recombinant human nerve growth factor eye drop (Cenegermin) had been granted for marketing authorization by European Medicine Agency for the treatment of NK at stages 2 and 3 [4, 10]. Nerve growth factor eye drop is expected to be the most effective approach for the prevention and treatment of NK [4]. Amniotic membrane transplantation is also effective for treating PED associated with NK. In cases of PED caused by diabetic keratopathy, Mucosta eye drops with anti-inflammatory effect for suppressing cytokine production can improve the defects [11]. In the present case, the epithelial defects were improved to a certain level with oil eye ointment and Mucosta eye drops. However, SPK, corneal superficial neovascularization, and corneal stromal edema persisted for several months after surgery. Because the pain of postherpetic neuralgia that had persisted for 8 years disappeared immediately after the trigeminal ganglion block, the patient was greatly relieved and satisfied despite decreased VA. In this case, the epithelial defects ameliorated relatively soon and did not progress to corneal erosion. Because the effect of the trigeminal ganglion nerve block does not last permanently, we closely followed up the patient to observe any signs of corneal opacity caused by infection and/or ulceration. The patient's corneal sensation gradually recovered and the ocular findings also showed improvement. The cornea eventually recovered without leaving any damage.

In conclusion, when corneal epithelial defects persist and recovery takes time, special care should be given until the corneal sensation ameliorates, such as replenishing treatment with SP + IGF-1 and recombinant human nerve growth factor eye drops to prevent the defects from deteriorating to PED, corneal erosion, or corneal perforation.

## Figures and Tables

**Table 1:** Details of the clinical findings of the right eye.

Time point after trigeminal nerve block	Clinical findings	Stage of NK	Corneal sensation (mm)
5 days: First visit	Severe conjunctival hyperemia Corneal superficial neovascularization Corneal epithelial defect Descemet's membrane folds Mild stromal edema	Stage 2	<10

1 week	Severe conjunctival hyperemia Corneal superficial neovascularization Corneal epithelial defect Cloudy and irregular corneal epithelium Descemet's membrane folds	Stage 2	<10

1 month	Superficial punctate keratopathy (SPK) Severe conjunctival hyperemia Corneal superficial neovascularization Corneal stromal edema	Stage 2	10

2 months	Superficial punctate keratopathy (SPK) Conjunctival hyperemia Corneal superficial neovascularization Irregularity of corneal epithelium Stromal scarring in the central cornea	Stage 1-2	10

5 months	Mild irregularity of corneal epithelium, stromal scarring in the central cornea, conjunctival hyperemia and corneal superficial neovascularization are overall improving.	Stage 1	50

6 months	SPK and conjunctival hyperemia, corneal superficial neovascularization, and corneal stromal edema were all ameliorated.		60
